# Supplementary material for: Intranasal type I interferon treatment is beneficial only when administered before clinical signs onset in the SARS-CoV-2 hamster model
Source: PLoS Pathog. 2021 Aug 9;17(8):e1009427. doi: 10.1371/journal.ppat.1009427 (PMC8376007; doi:10.1371/journal.ppat.1009427)
Supplement: S1 Table — (DOCX) [file ppat.1009427.s001.docx]

S1 Table. List of primers

| **Gene name** | **Primers sequences (5’ to 3’)** | **NCBI accession number or Reference** |
| --- | --- | --- |
| CCL5 | ACTGCCTCGTGTTCACATCA | XM_005076936.3 |
|  | CCTTCGGGTGACAAAAACGA |  |
| CXCL10 | GCCATTCATCCACAGTTGACA | [1] |
|  | CATGGTGCTGACAGTGGAGTCT |  |
| IFN-α7 | CTGGTGGCTGTGAGGAAATA | [2] |
|  | AGCAAGTTGGCTGAGGAAGA |  |
| IFN-γ | GGCCATCCAGAGGAGCATAG | [3] |
|  | TTTCTCCATGCTGCTGTTGAA |  |
| IL-1β | GGCTGATGCTCCCATTCG | [4] |
|  | CACGAGGCATTTCTGTTGTTCA |  |
| IL-6 | CCTGAAAGCACTTGAAGAATTCC | [1] |
|  | GGTATGCTAAGGCACAGCACACT |  |
| IL-10 | GTTGCCAAACCTTATCAGAAATGA | [4] |
|  | TTCTGGCCCGTGGTTCTCT |  |
| IL-12 | GGCCTTCCCTGGCAGAA | [1] |
|  | ATGCTGAAAGCCTGCAGTAGAAT |  |
| ISG15 | AAAGCCTACAGCCATGACCT | XM_013119951.2 |
|  | TTAGTCAGGGGCACCAGGAA |  |
| Mx1 | GCGCTTCCAGACTCTTCTGA | XM_021229467.1 |
|  | CCTAAGATACATGCGATGGCG |  |
| Mx2 | CCAGTAATGTGGACATTGCC | [3] |
|  | CATCAACGACCTTGTCTTCAGTA |  |
| OAS3 | AGGTGCTTAAGGTGGTTAAGGG | [5] |
|  | TGCTCAGAGAAGTGCTGGAAG |  |
| RPL18 | GTTTATGAGTCGCACTAACCG | [3] |
|  | TGTTCTCTCGGCCAGGAA |  |
| RPS6KB1 | TCAGACCGGTGGAAAACTCTAC | [5] |
|  | TGATGCAAATGCCCCAAAGC- |  |
| SARS-CoV-2 TRS-L | CTCTTGTAGATCTGTTCTCTAAACGAAC | [6] |
| SARS-CoV-2 TRS-N | GGTCCACCAAACGTAATGCG |  |
| TNF-α | GGAGTGGCTGAGCCATCGT | [1] |
|  | AGCTGGTTGTCTTTGAGAGACATG |  |

References

1. Marzi A, Banadyga L, Haddock E, Thomas T, Shen K, Horne EJ, et al. A hamster model for Marburg virus infection accurately recapitulates Marburg hemorrhagic fever. Sci Rep. 2016;6: 39214. doi:10.1038/srep39214

2. Schountz T, Campbell C, Wagner K, Rovnak J, Martellaro C, DeBuysscher BL, et al. Differential Innate Immune Responses Elicited by Nipah Virus and Cedar Virus Correlate with Disparate In Vivo Pathogenesis in Hamsters. Viruses. 2019;11. doi:10.3390/v11030291

3. Zivcec M, Safronetz D, Haddock E, Feldmann H, Ebihara H. Validation of assays to monitor immune responses in the Syrian golden hamster (Mesocricetus auratus). J Immunol Methods. 2011;368: 24–35. doi:10.1016/j.jim.2011.02.004

4. Atkins C, Miao J, Kalveram B, Juelich T, Smith JK, Perez D, et al. Natural History and Pathogenesis of Wild-Type Marburg Virus Infection in STAT2 Knockout Hamsters. J Infect Dis. 2018;218: S438–S447. doi:10.1093/infdis/jiy457

5. Toth K, Lee SR, Ying B, Spencer JF, Tollefson AE, Sagartz JE, et al. STAT2 Knockout Syrian Hamsters Support Enhanced Replication and Pathogenicity of Human Adenovirus, Revealing an Important Role of Type I Interferon Response in Viral Control. PLoS Pathog. 2015;11: e1005084. doi:10.1371/journal.ppat.1005084

6. Yang L, Han Y, Nilsson-Payant BE, Gupta V, Wang P, Duan X, et al. A Human Pluripotent Stem Cell-based Platform to Study SARS-CoV-2 Tropism and Model Virus Infection in Human Cells and Organoids. Cell Stem Cell. 2020;27: 125-136.e7. doi:10.1016/j.stem.2020.06.015
