# Supplementary figures and images for: Intranasal type I interferon treatment is beneficial only when administered before clinical signs onset in the SARS-CoV-2 hamster model
Source: PLoS Pathog. 2021 Aug 9;17(8):e1009427. doi: 10.1371/journal.ppat.1009427 (PMC8376007; doi:10.1371/journal.ppat.1009427)

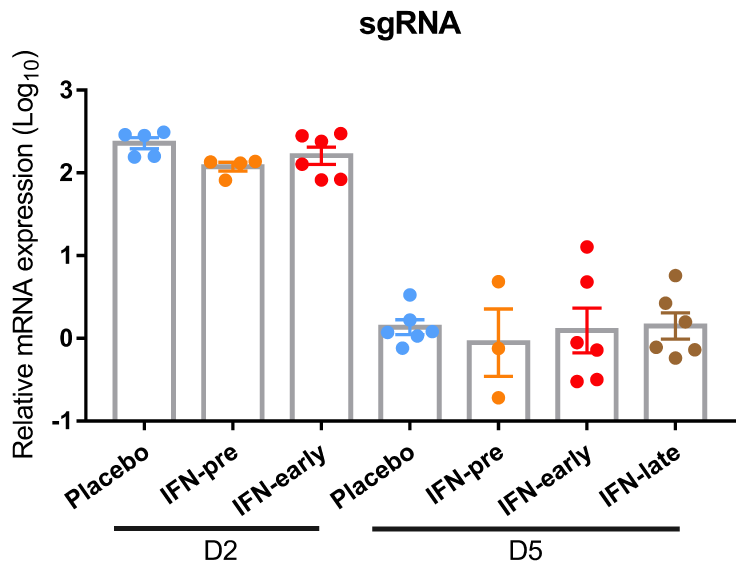

Supplement: S1 Fig — Nasal turbinates were harvested at day 2 post-infection (D2) or day 5 post-infection (D5). Viral sgRNA levels relative to the housekeeping genes RPL18 and RPS6KB1 were determined by RT-qPCR. Results are expressed as means ± SEM. Statistical analysis: one-way ANOVA with Tukey’s multiple comparisons test. (TIF) [file ppat.1009427.s002.tif]

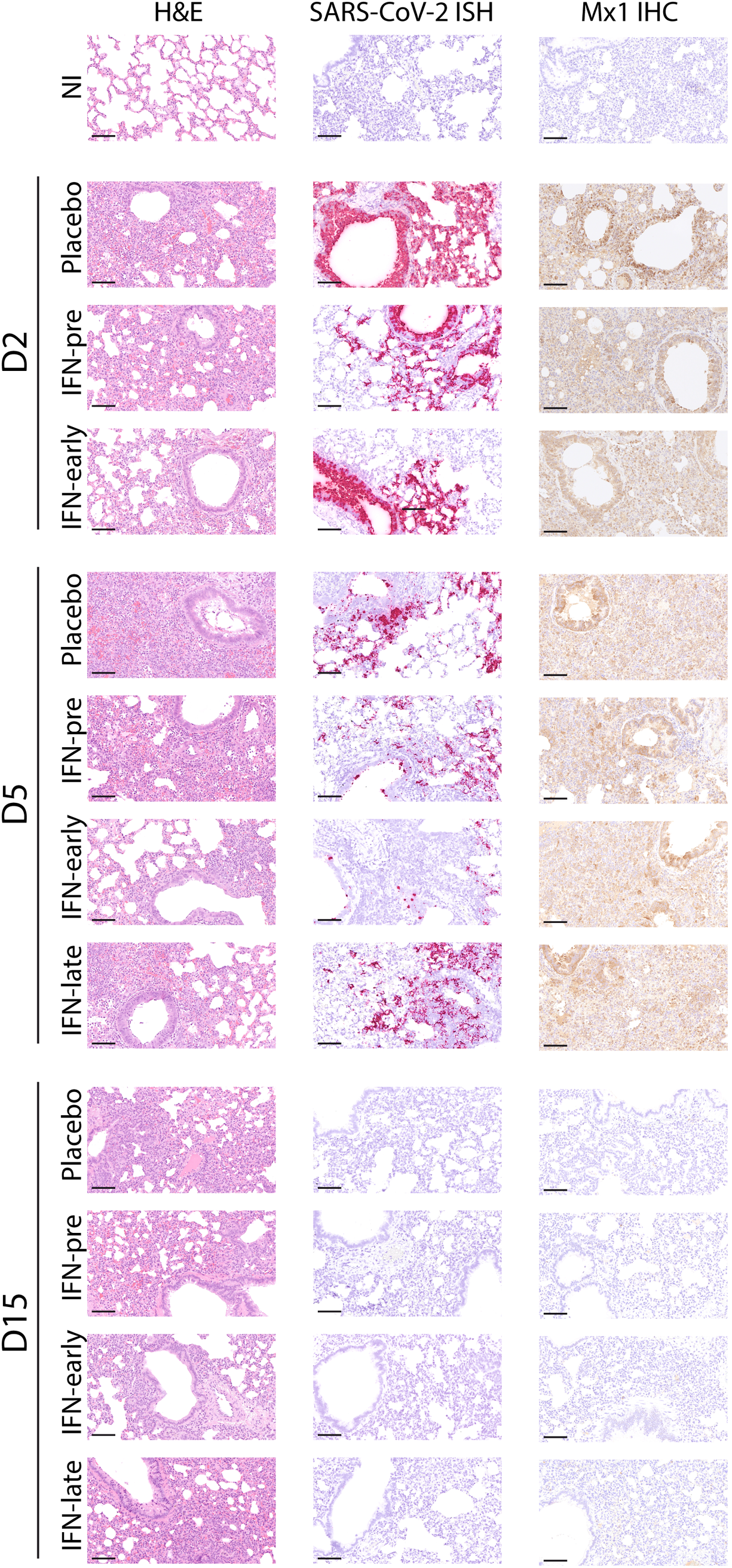

Supplement: S2 Fig — Representative pictures were selected to display the pathology from haematoxylin and eosin (H&E) stained lung section, viral RNA in lung sections stained with RNAScope in situ hybridization (ISH) and Mx1 protein detected by immunohistochemistry (IHC). D2: day 2 post infection; D5: day 5 post infection; D15: day 15 post infection. Scale bar: 100μm. (TIF) [file ppat.1009427.s003.tif]

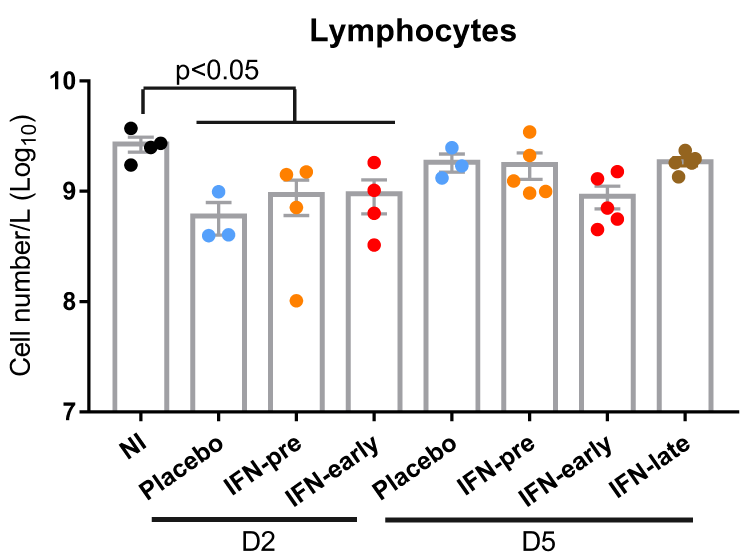

Supplement: S3 Fig — A complete blood count analysis was performed as described in the methods section. D2: day 2 post infection; D5: day 5 post infection. Results are expressed as means ± SEM. Statistical analysis: one-way ANOVA with Tukey’s multiple comparisons test. (TIF) [file ppat.1009427.s004.tif]

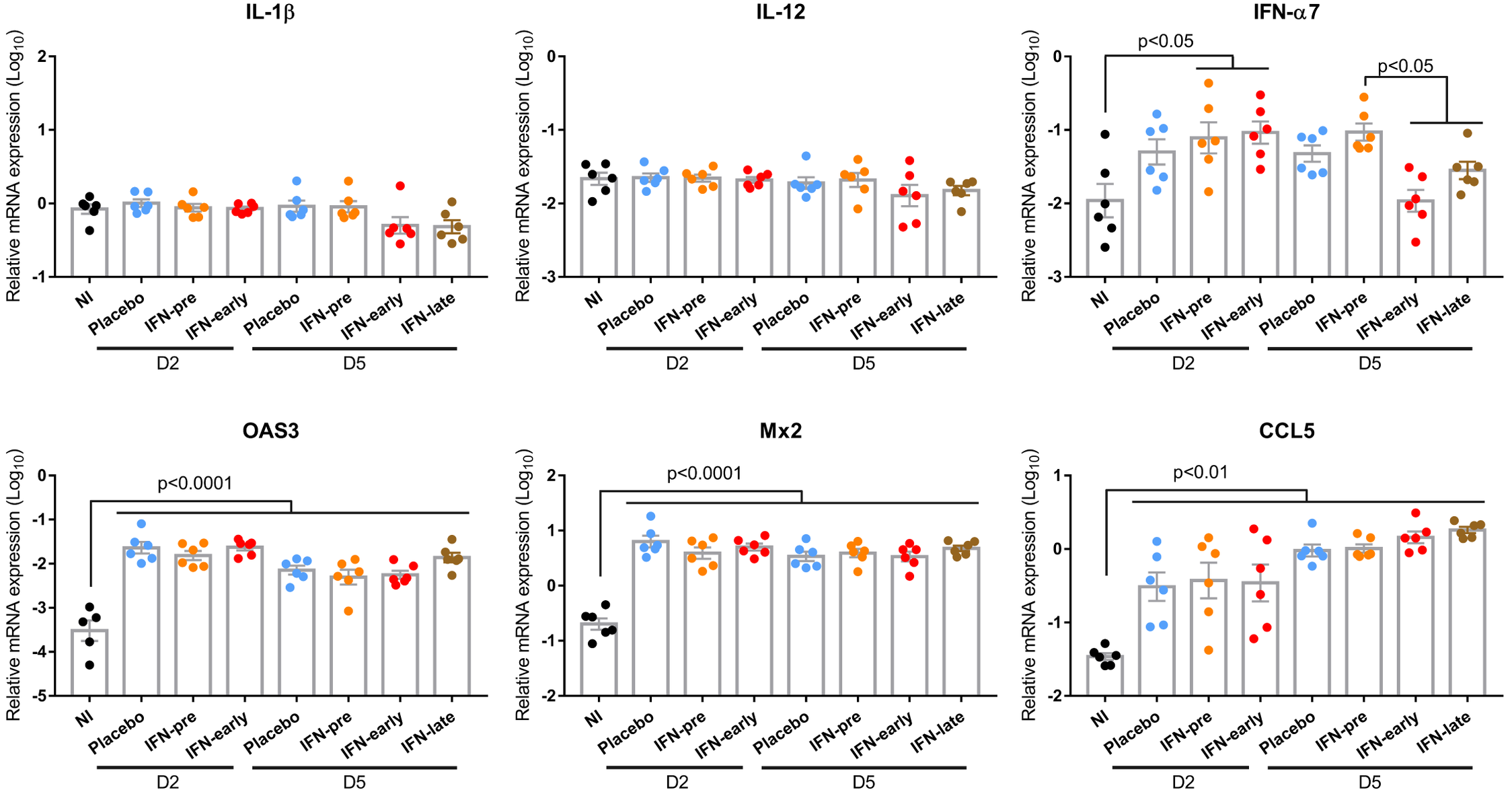

Supplement: S4 Fig — Lung transcripts levels of IL-1β, IL-12, IFN-α7, OAS3, Mx2, CCL5 relative to the housekeeping genes RPL18 and RPS6KB1 determined by RT-qPCR. D2: day 2 post infection; D5: day 5 post infection; D15: day 15 post infection. Results are expressed as means ± SEM. Statistical analysis: one-way ANOVA with Tukey’s multiple comparisons test. (TIF) [file ppat.1009427.s005.tif]

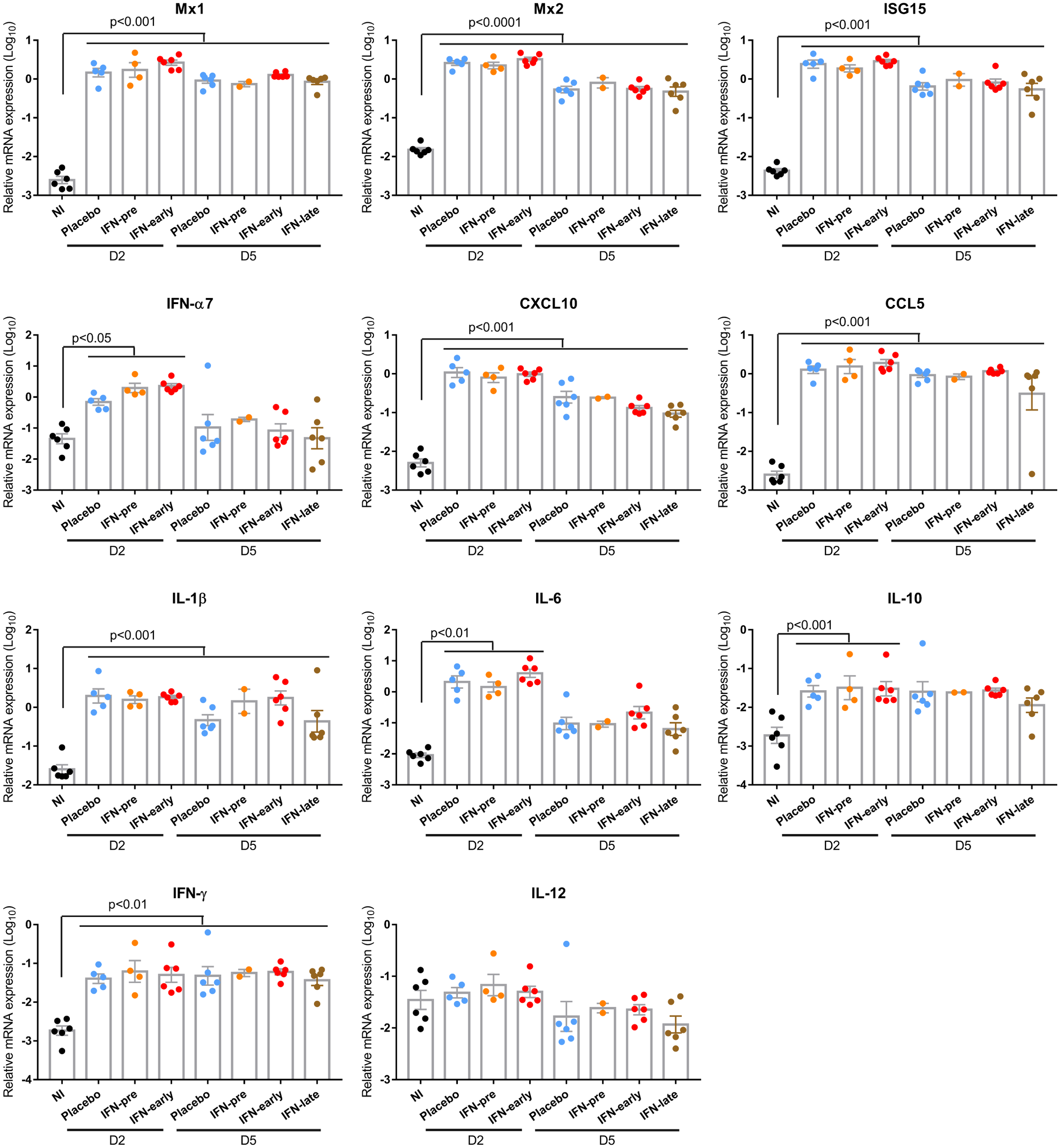

Supplement: S5 Fig — Nasal turbinates transcripts levels of Mx1, Mx2, ISG15, IFN-α7, CXCL10, CCL5, IL-1β, IL-6, IL-10, IFN-γ and IL-12 relative to the housekeeping genes RPL18 and RPS6KB1 determined by RT-qPCR. D2: day 2 post infection; D5: day 5 post infection; D15: day 15 post infection. Results are expressed as means ± SEM. Statistical analysis: one-way ANOVA with Tukey’s multiple comparisons test. (TIF) [file ppat.1009427.s006.tif]

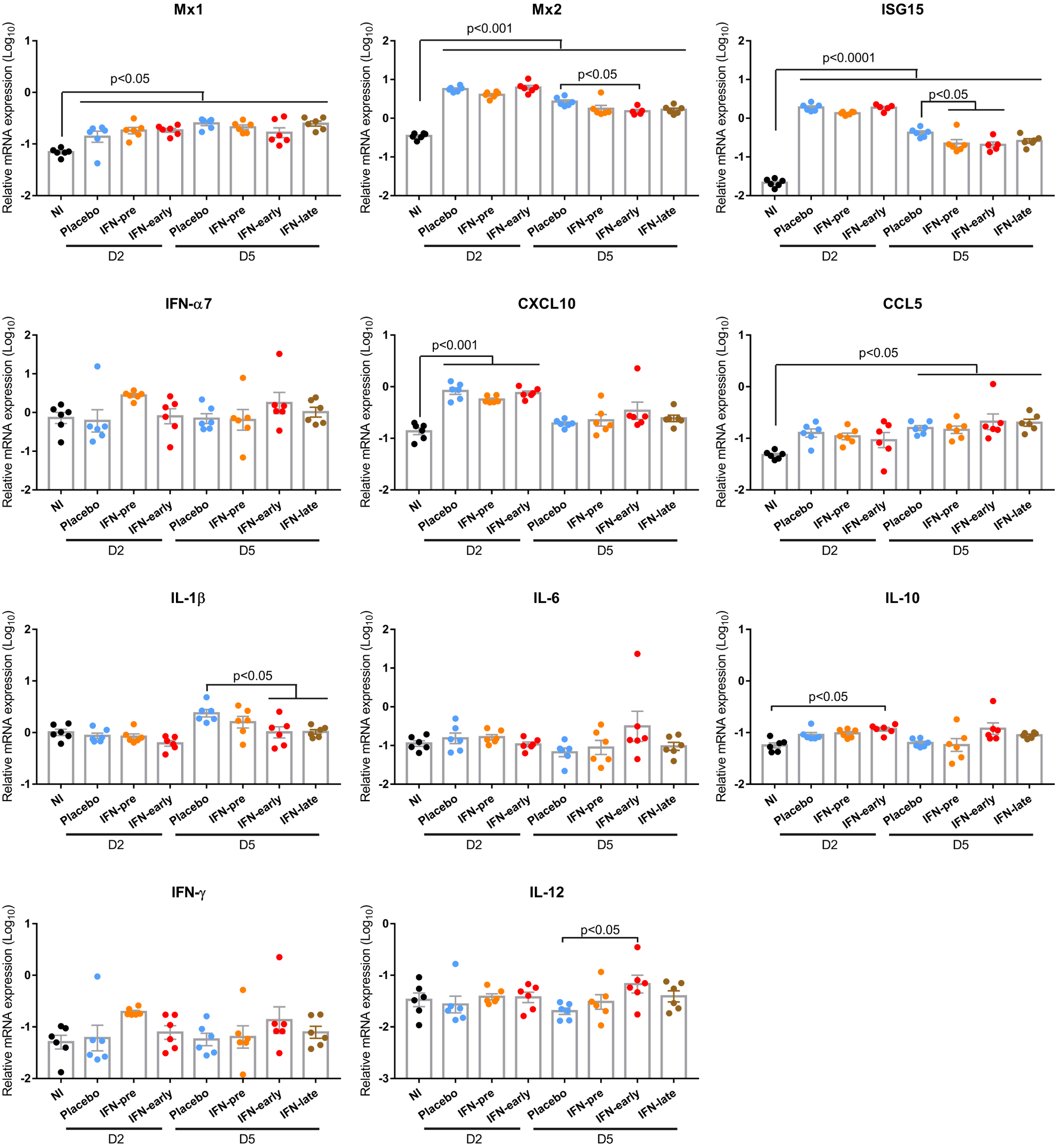

Supplement: S6 Fig — Spleen transcripts levels of Mx1, Mx2, ISG15, IFN-α7, CXCL10, CCL5, IL-1β, IL-6, IL-10, IFN-γ and IL-12 relative to the housekeeping genes RPL18 and RPS6KB1 determined by RT-qPCR. D2: day 2 post infection; D5: day 5 post infection; D15: day 15 post infection. Results are expressed as means ± SEM. Statistical analysis: one-way ANOVA with Tukey’s multiple comparisons test. (TIF) [file ppat.1009427.s007.tif]

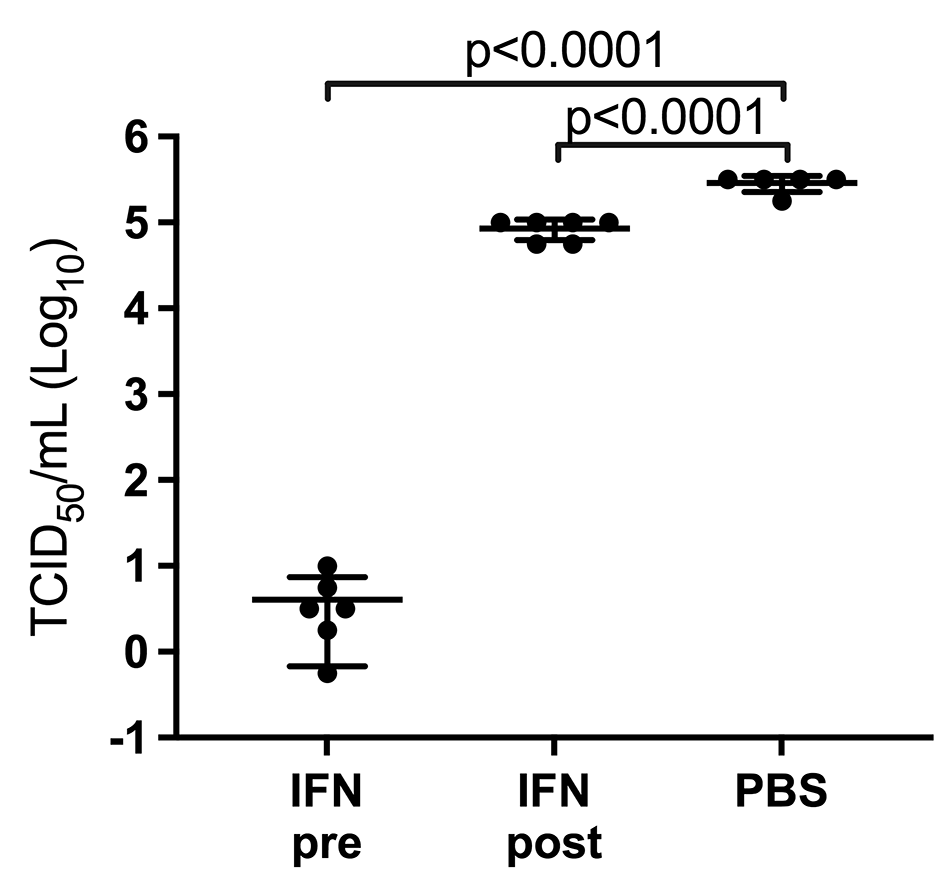

Supplement: S7 Fig — Vero-E6 cells were treated either with placebo or with 103 UI/mL recombinant universal IFN-α 18 hours prior to infection (IFN-pre) or 6 hours post infection (IFN-post). Equivalent volume of PBS was used as a negative control. Viral titers were determined by TCID50 from supernatants collected 24 hours post infection. Each dot represents a technical replicate of a representative experiment performed twice. Results are expressed as means ± SEM. Statistical analysis: one-way ANOVA with Tukey’s multiple comparisons test. (TIF) [file ppat.1009427.s008.tif]
