# Supplementary material for: Intranasal type I interferon treatment is beneficial only when administered before clinical signs onset in the SARS-CoV-2 hamster model
Source: PLoS Pathog. 2021 Aug 9;17(8):e1009427. doi: 10.1371/journal.ppat.1009427 (PMC8376007; doi:10.1371/journal.ppat.1009427)
Supplement: Attachment — Submitted filename: Reply to reviewers.docx [file ppat.1009427.s009.docx]

**Romain Volmer**

UMR INRAE-ENVT

Interactions hôtes-agents pathogènes (IHAP)

Email: romain.volmer@envt.fr

Toulouse, June 4 2021

Dear Editor, Dear Reviewers

Thanks to you and the three colleagues for reviewing our manuscript “Intranasal type I interferon treatment is beneficial only when administered before clinical signs onset in the SARS-CoV-2 hamster model” by Bessière et al., for which we provide a revised version.

As an overview, we should like to note that further experiments were conducted to demonstrate that intranasal type I IFN treatment in non-infected hamsters was able to induce Mx1 protein expression in the pulmonary cells targeted by SARS-CoV-2. In addition, we provide evidence that intranasal type I IFN treatment every other day was able to maintain a high level of Mx1 protein expression in the lung. Thus, the observed limited consequences of type I IFN treatment on viral replication are not due to an inadequate stimulation of type I interferon stimulated gene expression in our experiments.

In addition, as requested by PLoS Pathogens editorial staff, we have formatted the revised manuscript to include the manuscript sections “Abstract, Author summary, Introduction, Results, Discussions, Methods”.

Please find below a point-by-point response to the editor and reviewers comments.

**Editor’s comments:**

We thank the Editor for considering our manuscript as a sound addition to the SARS-CoV-2 field. The TenOever laboratory published a comprehensive study on the immune response to SARS-CoV-2 infection in hamsters and on the consequences of prophylactic type I IFN treatment on SARS-CoV-2 replication and immune response. Our study is different from the TenOever study as it was specifically designed to test how the timing of type I IFN treatment affected the efficacy against SARS-CoV-2. In addition to the prophylactic treatment, we have therefore evaluated two additional treatment regimens: treatment initiated one day post-infection and treatment initiated three days post-infection. In our opinion, this is a major addition. Indeed, prophylactic treatment may be potentially useful in very specific clinical or operational settings. However, the main objective in the current COVID-19 pandemic is to treat infected patients and therefore identifying when type I IFN is efficient and when it is not, is a sensible objective, in our opinion. We should like to stress that, to our knowledge, our study is the only one investigating how the timing of type I IFN treatment affects their efficacy against SARS-CoV-2. We therefore provide the scientific community with unique data of prime importance for the interpretation and design of clinical trials testing type I IFN treatment in COVID-19 patients.

(a) The key findings of our study are that prophylactic and early intranasal type I IFN treatments are able to significantly reduce weight loss, while treatment initiated at day three post-infection, when hamsters exhibited clinical signs, was inefficient. These conclusions are supported by valid statistical analyses, demonstrating that the number of animals used in this study was sufficient to achieve its main objectives. In our opinion, increasing the number of animals would neither change the above conclusions, nor change our interpretation on the modest effect of type I IFN treatments on viral titers in our experimental settings. For these reasons, our institutional ethics committee has advised against repeating experiments.

(b) We have provided further detail on the Syrian Hamster cytokines MilliPlex xMAP assay developed in collaboration with Merck Millipore in the Material and Methods section. The kit has an internal reference number (SPRCUS1249) and is available upon request from the corresponding author.

(c) As described above, we have performed additional experiments to demonstrate that intranasal type I IFN treatment in non-infected hamsters was able to induce the protein expression of the type I interferon-stimulated gene Mx1 in the pulmonary cells targeted by SARS-CoV-2.

(d) The discussion now incorporates a paragraph (lines 159-178 of the Manuscript) describing how type I IFN treatments exhibit a significant antiviral effect in some experimental conditions and not in others. In particular, a comparison with the TenOever study suggests that the observed differences could relate to the fact that the TenOever laboratory used a low dose SARS-CoV-2 inoculum (10^2^ pfu) and provided no data on weight loss, while we have used 10^4^ TCID_50_, an inoculum associated with significant weight loss in placebo-treated animals. The antiviral effects of type I IFN may be easier to observe when a low dose inoculum is used. This hypothesis is also supported by earlier studies in MERS-CoV infected mice, now referenced in the discussion. The authors would also like to point out that early findings from clinical trials of type III IFN treatments in COVID-19 patients showed little or no clinical benefit, although type III IFN was suggested to be a promising therapeutic option in SARS-CoV-2-infected mice (PMID: [33785743](https://pubmed.ncbi.nlm.nih.gov/33785743/), [32854108](https://pubmed.ncbi.nlm.nih.gov/32854108/)). It is thus important to provide the scientific community with experimental data on the consequences of intranasal type I IFN in different experimental settings, which will undoubtedly increase our knowledge on the interaction between SARS-CoV-2 and the innate immune response.

**Reviewer 1’s comments:**

- We thank reviewer 1 for his careful assessment of our manuscript. We agree that the lack of major effect of prophylactic type I IFN treatment on viral load is unexpected, given the high sensitivity of SARS-CoV-2 to type I IFN pre-treatment in cell culture (Fig. S7) and because the TenOever study provided evidence for a 1 Log_10_ reduction in viral titers in SARS-CoV-2 infected hamsters treated with type I IFN intranasally one day before infection. As stated lines 10-15 of this Letter, we now provide evidence that the innate immune response is induced by intranasal type I IFN treatment in the target cells of SARS-CoV-2 in the lung. The lack of effect or low effect of type I IFN treatment on viral load in our experiments is thus most likely attributable to the fact that we used a higher dose of SARS-Cov-2, in an effort to develop a more severe animal model and induce significant weight loss in hamsters (see Letter line 51-64). As SARS-CoV-2 expresses a broad array of antagonists of the innate immune response, we thus hypothesize that a high inoculum dose allows the virus to overcome the innate antiviral immune restriction and to replicate to some extent despite exogenous type I IFN treatments.

- We have provided further information in the *Materials and Methods* section on the Syrian Hamster cytokines Milliplex assay, which has been developed under a commercial agreement with Merck Millipore (see Letter lines 45-47). We agree that this assay will be very valuable to better understand SARS-CoV-2 pathogenesis in the Syrian hamster model and it will be available to the research community upon request.

- We have not repeated the experiment for ethical and scientific reasons, as we believe that our conclusions are valid and would not change by repeating the experiment (see Letter line 37-44).

Minor comments:

- ”78” referred to the number of animals and not their age. We modified the sentence to make this clearer.

- We have added information on the source and dosage of type I IFN in the main text.

**Reviewer 2’s comments:**

- We thank reviewer 2 for considering that our manuscript provides new details concerning the timing or type I IFN treatment that are important to the field. Indeed, this is the main objective of this work and thus complements previous work in hamsters by the TenOever laboratory and other studies in mice, which have solely investigated prophylactic or very early treatments with type I IFN, type III IFN or poly(I:C). We thank Reviewer 2 for appreciating the usefulness of our study in regards to design and interpretation of clinical trials testing type I IFN treatment in COVID-19 patients.

- We have changed the representative pictures in the manuscript (Fig. 3 and Fig. S2) to provide high magnification images illustrating lesions, viral RNA and Mx1 staining pattern. Due to the current unavailability of antibodies detecting the various Syrian hamster cell types, the nature of the cell infiltrates could only be assessed by H&E staining.

**Reviewer 3’s comments:**

- We thank reviewer 3 for evaluating our manuscript. We agree that the TenOever study provides important information on SARS-CoV-2 pathogenesis in Syrian hamsters, most particularly through the analysis of transcripts by RNA sequencing. However, the TenOever study only investigated prophylactic type I IFN treatment against SARS-CoV-2. We designed our study to specifically investigate how the timing of the type I IFN treatment modulated its efficacy against SARS-CoV-2. We thus believe that our study provides very valuable information that, to our knowledge, are currently not found in any other study in animal models of SARS-CoV-2 infection and which are relevant to the treatment of COVID-19 patients (see also Letter lines 25-35).

- Our objective was not to suggest type I IFN as a therapeutic option against SARS-CoV-2, as it has been already tested in COVID-19 patients and is still currently being tested in a number of clinical trials. Our objective was to provide data that would help interpret clinical trials and, if possible, update the design of future clinical trials based on the timing of treatment initiation.

**Part II - Major Issues: Key Experiments Required for Acceptance**

**Reviewer #1:** We have performed additional experiments to analyse the location and intensity of the antiviral immune response in non-infected hamsters treated with type I IFN (see Letter lines 10-15).

**Reviewer #2:** We provide evidence that Mx1 protein expression remained stable for 48 hours post-treatment (see Manuscript lines 83-86). We therefore don’t think that prophylactic treatment would have been more effective if daily dosing was used instead of every two-day dosing. In addition, it is important to note that as soon as two days post-infection, the levels of ISGs were equivalent in placebo-treated and type I IFN-treated animals, indicating that ISGs had reached their maximum in response to virus-induced endogenous type I and type III IFNs production and could not be further augmented following exogenous type I IFN administration.

Figure 2 (now referenced as Figure 3): we had shown lung section pictures from day 5 post-infection in Figure 2 because this corresponds to the peak of clinical signs and because all experimental groups could be represented at day 5 (we have no late treatment animals at day 2 post-infection because “IFN-late” treatment started at day 3 post-infection). However, we agree that the most significant differences in lesions have been observed at day 2. We have therefore modified Figure 2 (now referenced as Figure 3) to show lung section pictures at a high magnification from day 2 post-infection (Fig. 3). In addition, all the microscopic pictures have been switched to high magnification images illustrating lesions, viral RNA and Mx1 staining pattern and are provided for all experimental groups and days-post-infection (Fig. S2).

The strongest reduction in viral titers in the lungs was observed at day 5 post-infection. In the nasal turbinates, viral RNA levels are strongly reduced in all groups at day 5 compared to day 2 post-infection. The inability to detect a reduction in nasal turbinates viral RNA levels upon type I IFN treatment could be due to the fact that the viral RNA levels are already low in nasal turbinates, whereas viral titers remained high in the lungs of placebo-treated animals at day 5 post-infection. Interestingly, a similar observation was made in SARS-CoV-2 infected mice treated with type III IFN: a significant reduction of viral titers was observed in the lungs of mice treated with type III IFN, while no significant reduction of viral titers was observed in the nasal turbinates, and this was associated with high titers in the lung and low titers in the nasal turbinates of placebo-treated mice (Figure 4 in PMID: [32854108](https://pubmed.ncbi.nlm.nih.gov/32854108/); Ref (25) in the manuscript).

**Reviewer #3:** We have performed additional experiments to analyse the level of Mx1 mRNA and protein in hamsters treated with 7.10^5^IU IFN compared to 10^5^IU IFN. While the levels of Mx1 mRNA was similar, we observed an increase in the percentage of Mx1 positive lung in animals administered 7.10^5^ compared to 10^5^IU IFN. However, we chose the dose of 10^5^IU IFN per hamster, because it corresponds to the dosage used in human clinical trials (see Manuscript lines 88-92 and 234-238 for details on the hamster equivalent dosage calculations).

Quantification of lung areas was performed using state-of-the art methodologies by an experienced pathologist. We are therefore confident that the modest differences do indeed correspond to modest effects of the type I IFN treatments.

**Part III - Minor Issues**

**Reviewer #2:**

- Figure 1B (now referenced as Figure 2B): Yes, weight loss is significantly different between placebo and IFN-pre and between placebo and IFN-early (p<0.001; weight loss was statistically analyzed using two-way ANOVA with Geisser-Greenhouse correction followed by Tukey’s multiple comparisons test (GraphPad Prism Software, USA) with the p values indicated for the weight loss corresponding to the results of Tukey’s post hoc tests). We have modified the figure to make this clearer.

- We have modified figures 1C, 1D and 2E (now referenced as Figures 2C, 2D and 2E) to indicate oropharyngeal swabs and lungs as recommended.

- We have modified the manuscript to clearly indicate that only IFN-early treatment led to a significant reduction in the histopathology scores (Figure 3B): line 118 in the manuscript.

**Reviewer #3:** see lines 45-47 of this letter
