# Supplementary material for: Intranasal type I interferon treatment is beneficial only when administered before clinical signs onset in the SARS-CoV-2 hamster model
Source: PLoS Pathog. 2021 Aug 9;17(8):e1009427. doi: 10.1371/journal.ppat.1009427 (PMC8376007; doi:10.1371/journal.ppat.1009427)
Supplement: Attachment — Submitted filename: Reply to reviewers round #2.docx [file ppat.1009427.s010.docx]

**Romain Volmer**

UMR INRAE-ENVT

Interactions hôtes-agents pathogènes (IHAP)

Email: romain.volmer@envt.fr

Toulouse, July 6 2021

Dear Editor, Dear Reviewers

Thanks to you and the three colleagues for reviewing our manuscript “Intranasal type I interferon treatment is beneficial only when administered before clinical signs onset in the SARS-CoV-2 hamster model” by Bessière et al., for which we provide another revised version.

Please find below our response to Reviewers’ comments.

**Reviewer 1’s comments:**

We thank Reviewer 1 for his comments on the manuscript and appreciating the improvements provided by the additional experiments performed. We thank Reviewer 1 for suggesting that we should use a non-parametric test for the analysis of H&E scores in Figure 3B. We agree and have analysed these data using the non-parametric Mann-Whitney test and have modified the figure legend accordingly. Please note that the p vales remain p<0.05 and p<0.01.

**Reviewer 2’s comments:**

We thank Reviewer 2 for his comments on the manuscript and for writing that “the authors do not overstate their findings and accurately present them as part of our growing understanding of how to treat COVID-19 patients”.

**Reviewer 3’s comments:**

We thank Reviewer 3 for his comments on the manuscript. Reviewer 3 may have not seen the marked-up manuscript beginning on page 42 of the revised submission (PPATHOGENS-D-21-00452R1). We have not changed the title and believe that the title does not overstate our findings, since we present them in the context of the Syrian hamster model.

Major comments:

We thank Reviewer 3 for his comments on the manuscript. As stated in the round 1 reply to Reviewers, our results are supported by valid statistical analyses, demonstrating that the number of animals used in this study was sufficient to achieve its main objectives. We believe that increasing the number of animals would neither change our conclusions, nor change our interpretation on the modest effect of type I IFN treatments on viral titers in our experimental settings. For these reasons, our institutional ethics committee has advised against repeating experiments.

Minor comments:

The Materials and Methods section now incorporates a paragraph providing more details about the semi quantitative scoring that was applied to evaluate the lung lesions (see lines 290-299 of the revised Manuscript).

Regarding the Milleplex assay, we certify that we have provided all the information that the Merck-Millipore company has agreed to share with us. As described in the Material and Methods section, the kit has an internal reference number (SPRCUS1249) and was developed by combining commercial assays for rat (RECYTMAG-65K) and feline (FCYTMAG-20K) species.
